# An evolutionary game analysis of supervision behavior in public-private partnership projects: Insights from prospect theory and mental accounting

**DOI:** 10.3389/fpsyg.2022.1023945

**Published:** 2023-01-11

**Authors:** Xiaotong Cheng, Min Cheng

**Affiliations:** Department of Management Science and Engineering, School of Management, Shanghai University, Shanghai, China

**Keywords:** PPP projects, supervision, prospect theory, mental accounting, evolutionary game

## Abstract

Effective supervision is one of the important ways to ensure the smooth implementation of Public-Private Partnership (PPP) projects. To understand the characteristics of the decision-making behavior of the public and private sectors in the supervision of PPP projects and the influencing mechanisms of some factors, we combine prospect theory and mental accounting theory into the evolutionary game analysis. First, we use prospect theory to reflect the behavioral characteristics of game players when making decisions and classify the value function into a valence account and a cost account according to the mental accounting theory. Accordingly, we construct a payoff matrix based on prospect theory and mental accounting theory, and the system’s equilibrium state is analyzed. Then, based on numerical simulations, the influence of different parameters on the behavior of the public and private sectors is analyzed, and management suggestions for practical reference are put forward based on the simulation results. The results show that the greater the perceived cost of active behavior for the public and private sectors, the less likely they will take active behavior. Secondly, there is insufficient incentive for the private sector to fulfill contracts when the penalties for its opportunistic behavior are minor. Thirdly, increasing the cost reference points and decreasing the valence reference points will promote the public and private sectors to adopt active behavior. Fourth, the public sector and the private sector are more inclined to take active behavior when they need to bear more significant risk losses. This study provides new ideas for the analysis of the game players’ decision-making behaviors in the supervision of PPP projects and delivers a decision-making reference for reasonable supervision.

## 1. Introduction

Public-Private Partnership (PPP) is a long-term contractual agreement between the public and private sectors to deliver infrastructure or provide public services (Amadi et al., 2018; Hadi and Erzaij, 2019). In PPP projects, the private sector usually undertakes works, such as design, construction, financing, and operation and gains profits by providing services during the operation stage (Amadi et al., 2020). The public sector is responsible for supervising the quality and price of the services provided by the private sector to ensure public interest (Kwak et al., 2009). In recent years, PPP has been widely used in infrastructure construction and public services provision in many countries because it can relieve the public sector’s financial pressure and improve the supply efficiency of public goods (Oliveros-Romero and Aibinu, 2019; Song et al., 2020; Liu et al., 2021). However, some PPP projects failed due to the opportunistic behavior of the private sector in pursuit of profit and the weak supervision of the public sector (Wan et al., 2015).

As the critical stakeholders of PPP projects, the public and private sectors have different expected benefits. The public sector expects the project to meet the public’s demands for public goods or services, while the private sector expects economic benefits from the project (Lohmann and Roetzel, 2014). The pursuit of profit by the private sector may make it opportunistic in the operation of PPP projects (Feng et al., 2021). For example, the private sector may reduce the frequency or standard of road maintenance to save operating costs in expressway PPP projects. Because the opportunistic behavior of the private sector may harm the public interest, the public sector needs to establish an effective supervision mechanism. In addition, the public nature of PPP projects also makes the public sector responsible for supervision. Ika (2015) analyzed 178 projects and found that supervision significantly affects project performance. But the supervision of the project is not always effective (Cao and Wang, 2022). The public sector may not be able to supervise effectively due to insufficient supervision capacity, unclear responsibilities, or consideration of supervision costs.

The different interest needs of the public and private sectors make them have game behaviors in the supervision of PPP projects. On the one hand, the private sector will weigh the pros and cons between the risk of violations and the economic benefits to make decisions about fulfilling contracts or opportunistic behavior. On the other hand, the public sector may also have a strategic choice of supervising actively or negatively. Meanwhile, both will adjust their behavioral strategies according to the other’s behavior. Exploring the evolutionary law of game behavior between the public and private sectors in the supervision of PPP projects can clarify the impact mechanism of influencing factors on game behavior and provide a basis for establishing a scientific and reasonable PPP project supervision mechanism. Therefore, we study the evolution and influencing factors of decision-making behaviors of the public sector and private sector in the supervision of PPP projects.

The rest of this paper is organized as follows. Section 2 reviews the relevant literature on supervision behavior in PPP projects. Then, based on prospect theory and mental accounting theory (PT-MA), an evolutionary game model of the public sector and private sector in the supervision of PPP projects is constructed in Section 3. Section 4 analyzes the interactive behaviors of the game players and the equilibrium state of the established evolutionary model. To more intuitively reflect the impact of different parameters on the behavioral choice of the public and private sector in PPP projects, numerical simulations are conducted, and the simulation results are explained in Section 5. Finally, the conclusions and relevant policy recommendations are presented in Section 6.

## 2. Literature review

Some scholars have pointed out that effective supervision is essential to successfully implementing PPP projects. For example, Yun et al. (2015) empirically analyzed the factors that affect the success of PPP projects. The results showed that the public sector’s supervision is one of the critical factors for the success of the projects. Sabry (2015) pointed out that effective supervision is helpful to the implementation of PPP projects. The importance of establishing an effective supervisory mechanism for PPP projects has been recognized.

There is a game between the public sector and the private sector in the supervision of PPP projects. Understanding their game relationship will help improve the supervisory mechanism. Game theory is a method for studying the behavior of game players (An et al., 2022). Some scholars have used the classical game theory to analyze the supervision behavior in PPP projects (Sabry, 2015; Ouenniche et al., 2016; Leong and Qian, 2018; Liang et al., 2019). The classical game theory assumes that game players are fully rational (Smith, 1996; Ng et al., 2013). However, it is difficult for the game players to be fully rational due to incomplete information, insufficient cognitive ability, and risk perception. Therefore, the assumption of full rationality is often not suitable for describing the behavior of game players in reality.

Evolutionary game theory assumes that players are bounded rationality, and they adjust their strategies based on the actions of other players, which is more consistent with the actual behavioral characteristics of the players (Zhu et al., 2022). Thus, the evolutionary game theory is more suitable for studying the supervision issues of PPP projects. Some scholars have used the evolutionary game method to study supervisory behavior in PPP projects. For example, Liu et al. (2017) studied the supervision behavior of the public sector and the opportunistic behavior of the private sector during the operation of PPP projects based on the evolutionary game method and put forward suggestions for the public sector’s supervision. Li et al. (2016) explored the impact of public participation on the supervision behavior of PPP projects on the basis of evolutionary game theory. Yue and Lin (2019) constructed an evolutionary game model to study the service quality supervision of pension PPP projects. Gao and Liu (2019) analyzed the choice of government supervision mode in the operation stage of the PPP project based on the evolutionary game. Li et al. (2022) constructed an asymmetric evolutionary game model to analyze the behavioral evolution law of government regulators and the private sector in water environment governance PPP projects and put forward regulatory suggestions for the public sector.

Although the assumption of bounded rationality is more realistic, the payoff matrix of the evolutionary game model in the above literature is constructed based on the expected utility theory. It assumes that the game players are risk neutral without reflecting the players’ perception of risk. The construction and operation process of PPP projects is complex. In the supervision of PPP projects, the decision-making behavior of public sector’s supervision and the private sector’s performing is often affected by subjective judgment and value perception. Tversky and Kahneman (1974) proposed prospect theory that fits the characteristics of human decision-making behavior based on cognitive psychology. Prospect theory argues that decision-makers value gains and losses differently, placing more emphasis on perceived gains than perceived losses. Some scholars have combined evolutionary game and prospect theory to study human behavior in different fields. For example, Shen et al. (2021) studied decision-making behaviors and influencing factors of local governments and polluting enterprises in watershed ecological compensation based on the evolutionary game theory and the prospect theory. Luo et al. (2018) introduced the prospect theory into evolutionary game analysis to construct a game model involving enterprises, consumers, and governments in managing food safety risks. They discussed the impact of influencing factors on their behavioral decisions. Chen et al. (2016) embedded the prospect theory into an evolutionary game framework to study the decision-making behaviors of pedestrians and drivers.

Although prospect theory incorporates the perceived value of decision makers, it still has certain limitations. Prospect theory aims to describe single-attribute decision-making problems but cannot reflect the situational dependence of decision-making behavior. Thaler (1985) proposed the mental accounting theory suitable for studying decision-makers’ behavior in a multi-attribute decision-making situation. Mental accounting is a cognitive bias that reflects people’s tendency to treat money differently according to its source or intended use (Moon et al., 1999). It means the decision-makers will evaluate gains and losses in two separate mental accounts. The expected utility in different mental accounts is different (Thaler, 1999).

Regarding the supervision of PPP projects, the public sector as the supervisor may actively supervise or not supervise well due to its limited supervision capacity or cost. The supervised private sector may fulfill contracts conscientiously due to considering penalties or act opportunistically because of economic benefits. The public and private sectors always make their own behavioral choices according to multiple factors and the other’s behavior. In other words, in the supervision of PPP projects, their choice of game behavior can be regarded as a multi-attribute decision-making problem. Thus, the combination of prospect theory and mental accounting can be applied to the study of decision-maker behavior in multi-attribute decision-making situations from the perspective of psychological perception. Therefore, the evolutionary game behavior of the public and private sectors in the supervision of PPP projects can be analyzed based on prospect theory and mental accounting theory to more truly describe decision-makers’ behavioral preferences and strategy choices under uncertainty.

## 3. Model assumptions and payoff matrix

### 3.1. Model assumptions

In the supervision of PPP projects, the public sector needs to ensure social benefits and consider the cost of supervision. The private sector may either act opportunistically in pursuit of profit or aggressively enforce contracts to avoid penalties due to opportunistic behavior. Whether the public sector actively supervises and the private sector operates in compliance is a dynamic evolutionary game process. The strategic choices of the two-game players interact with each other during the process of multiply dynamic evolutionary games. They will determine their optimal strategies and form a stable strategy (Friedman, 1998). To analyze the behavioral evolution mechanism of the public and private sectors in the supervision of PPP projects, we make the following assumptions.

#### 3.1.1. Assumption 1

There are two game players in the evolutionary game: the public sector and the private sector. As the supervisor of a PPP project, the public sector may supervise weakly due to unclear responsibilities and insufficient supervision capabilities. The private sector may act opportunistically driven by profits. Therefore, the strategies of the public sector and the private sector are denoted as $\left(A_{1},A_{2}\right)$ and $\left(B_{1},B_{2}\right)$, respectively. For the public sector, $A_{1}$ represents the strategy of strong supervision, and $A_{2}$ represents the strategy of weak supervision. The probability of the two strategies is a(0≤a≤1) and 1−a, respectively. For the private sector, $B_{1}$ represents the strategy of fulfilling contracts and $B_{2}$ represents the strategy of taking opportunistic behavior. The probability of the two strategies is b(0≤b≤1) and 1−b, respectively.

#### 3.1.2. Assumption 2

Both the public and private sectors are bounded rational in the game of PPP project supervision (Liu et al., 2017). Their decision-making behaviors come with features described by prospect theory, including certainty effect, reflection effect, loss aversion, small probability, and reference dependence (Kahneman and Tversky, 1979). The certainty effect means that people are risk averse when there is a sure prospect of returns. The reflection effect implies that people are risk averse when faced with a potential gain and risk-seeking when faced with a loss. Loss aversion means people prefer to minimize losses than maximize profits. Small probability means people tend to discount tiny possibilities despite possible adverse consequences. Reference dependence is a feature in which people evaluate outcomes in terms of current situations rather than final status as in expected utility theory (Fennema and Wakker, 1997; Chung et al., 2019). According to prospect theory, the overall utility of the decision depends on the value function and the probability weighting function (Senbil and Kitamura, 2004). The overall utility function V can be expressed as Equation (1).


(1) $$V=\overset{n}{\underset{i=1}{\sum }}\pi \left(p_{i}\right)v\left(x_{i}\right)$$

where $v\left(x_{i}\right)$ is the value function. $x_{i}$ is actual gains or losses of event i. $\pi \left(p_{i}\right)$ is the probability weighting function, which is a non-linearly increasing function of objective probability $p_{i}$ and satisfies π(0)=0 and π(1)=1. For small probabilities, π(p)>p. $\pi \left(p_{i}\right)$ can be calculated by Equation (2).


(2) $$\pi \left(p_{i}\right)=\frac{p_{i}{}^{r}}{{\left[p_{i}{}^{r}+{\left(1-p_{i}\right)}^{r}\right]}^{\frac{1}{r}}}$$

where r(0≤r≤1)is a parameter reflecting the curvature of the weighting function. The smaller ris, the more cured it is.

Since the public sector and the private sector have different perceptions of the gains and losses in the supervision of PPP projects, their value function is divided into a valence account and a cost account according to the theory of mental accounts, each with its own reference point. The value function based on PT-MA is expressed as follows:


(3) $$T\left(x\right)=\{{}_{-\lambda {\left(S_{0}-x\right)}^{\beta },x<S_{0}}^{{\left(x-S_{0}\right)}^{\alpha },x\geq S_{0}}$$

(4) $$Z\left(x\right)=\{{}_{-{\left(S_{1}-x\right)}^{\sigma },x<S_{1}}^{\delta {\left(x-S_{1}\right)}^{\varphi },x\geq S_{1}}$$

where T(x) is the value function of the valence account. Z(x) is the value function of the cost account. $S_{0}$ and $S_{1}$ are the reference points in the value function of the valence account and the cost account, respectively. λ and δ indicate game players’ sensitive degree of seeking gains and avoiding losses, respectively, (λ≥1, δ≥1). α and β are the risk preference coefficients in the value function of the valence account, which, respectively, reflect the risk attitudes of game players to the gains and losses with $S_{0}$ as the reference point (0<α,β<1). φ and σ are the risk preference coefficients in the value function of the cost account, which, respectively, reflect the risk attitudes of game players to the losses and gains with $S_{1}$ as the reference point (0<φ,σ<1).

#### 3.1.3. Assumption 3

There is information asymmetry between the public sector and the private sector. When the public sector strongly supervises the private sector and the private sector actively fulfills the contract, i.e., their strategy is $\left(A_{1},B_{1}\right)$, the project risk is low. When one game player adopts an active strategy, and the other adopts a negative strategy, that is, when the game player’s strategy is $\left(A_{1},B_{2}\right)$ or$\left(A_{2},B_{1}\right)$, the probability of risk occurrence will increase. When the public sector supervises weakly and the private sector acts opportunistically, i.e., their strategy is $\left(A_{2},B_{2}\right)$, the project risk is high.

#### 3.1.4. Assumption 4

When the public sector adopts a strong supervision strategy, its payoff (such as public praise, etc.) is $R_{1}$, and its perceived value of the cost paid is $I_{0}$. When the public sector adopts a weak supervision strategy, the perceived value of the cost is $I_{1}$ and the perceived value of the loss of credibility is $I_{2}\left(I_{1}+I_{2}<I_{0}\right)$. When the private sector fulfills the contract, its income is $R_{2}$, its perceived value of the cost is $C_{0}$, and the award from the public sector is Q. When the private sector act opportunistically, its income is $R_{3}$, the perceived value of cost is $C_{1}$, the perceived value of the loss of the private sector’s reputation is $C_{2}\left(C_{1}+C_{2}<C_{0}\right)$, and the penalty for breach of the contract imposed by the public sector is P.

#### 3.1.5. Assumption 5

The risk losses are shared by the public sector and private sector, and their risk loss is linearly related (Gao, 2015). When the game strategy is $\left(A_{2},B_{2}\right)$, the risk loss of the private sector is L, the joint risk loss of the public sector is hL, where h is the coefficient of joint risk loss. When the game strategy is $\left(A_{1},B_{2}\right)$, the risk loss of the private sector is $k_{1}L$, the joint risk loss of the public sector is $hk_{1}L$. When the game strategy is $\left(A_{2},B_{1}\right)$, the risk loss of the private sector is $k_{2}L$, the joint risk loss of the public sector is $hk_{2}L$. $k_{1}$ and $k_{2}$ are risk coefficients. The probability of a risk is q.

According to the above assumptions, the variables and parameters are summarized in Table 1.

**Table 1:** Variables and explanation.

Variables	Description
$R_{1}$	The payoff of the public sector when its strategy is $A_{1}$.
$R_{2}$	The income of the private sector when its strategy is $B_{1}$.
$R_{3}$	The income of the private sector when its strategy is $B_{2}$.
$I_{0}$	The perceived value of the cost by the public sector when its strategy is $A_{1}$.
$I_{1}$	The perceived value of the cost by the public sector when its strategy is $A_{2}$.
$I_{2}$	The perceived value of the credibility loss by the public sector when its strategy is $A_{2}$.
$C_{0}$	The perceived value of the cost by the private sector when its strategy is $B_{1}$.
$C_{1}$	The perceived value of the cost by the private sector when its strategy is $B_{2}$.
$C_{2}$	The perceived value of the loss of reputation by the private sector when its strategy is $B_{2}$.
*Q*	Rewards received by the private sector from the public sector.
P	The penalty imposed by the public sector when the private sector breaches contracts.
$k_{1}$	Risk coefficient when the public sector’s strategy is $A_{1}$ and the private sector’s strategy is $B_{2}$.
$k_{2}$	Risk coefficient when the public sector’s strategy is $A_{2}$ and the private sector’s strategy is $B_{1}$.
L	The risk loss of the private sector when the public sector’s strategy is $A_{2}$ and the private sector’s strategy is $B_{2}$.
*q*	Probability of risk.
*h*	Coefficient of joint risk loss.

### 3.2. Payoff matrix

Based on the above assumptions, a traditional payoff matrix is constructed, as shown in Table 2.

**Table 2:** Traditional payoff matrix based on the expected utility.

		Private sector	
		Fulfill contracts	Take opportunistic behavior
Public sector	Strong supervision	$R_{1}-I_{0}+Q,R_{2}-C_{0}+Q$	$R_{1}-I_{0}+P-hk_{1}qL,R_{3}-C_{1}-C_{2}-P-k_{1}qL$
	Weak supervision	$-I_{1}-I_{2}-Q-hk_{2}qL,R_{2}-C_{0}+Q-k_{2}qL$	$-I_{1}-I_{2}-hqL,R_{3}-C_{1}-C_{2}-qL$

Further, the payoff matrix of the two-game players based on PT-MA is shown in Table 3.

**Table 3:** Payoff matrix based on PT-MA.

		Private sector	
		Fulfill contracts	Take opportunistic behavior
Public sector	Strong supervision	$\begin{array}{l} T\left(R_{1}\right)-Z\left(I_{0}+Q\right),\\ T\left(R_{2}+Q\right)-Z\left(C_{0}\right) \end{array}$	$\begin{array}{l} T\left(R_{1}+P\right)-Z\left(I_{0}+hk_{1}\pi \left(q\right)L\right),\\ T\left(R_{3}\right)-Z\left(C_{1}+C_{2}+P+k_{1}\pi \left(q\right)L\right) \end{array}$
	Weak supervision	$\begin{array}{l} -Z(I_{1}+I_{2}+Q+hk_{2}\pi \left(q\right)L),\\ T\left(R_{2}+Q\right)-Z\left(C_{0}+k_{2}\pi \left(q\right)L\right) \end{array}$	$\begin{array}{l} -Z\left(I_{1}+I_{2}+h\pi \left(q\right)L\right),\\ T\left(R_{3}\right)-Z\left(C_{1}+C_{2}+\pi \left(q\right)L\right) \end{array}$

## 4. Model solution

### 4.1. Payoff function

The expected prospect values for the two strategies of the public sector in the supervision of a PPP project are represented as $V_{A1}$ and $V_{A2}$, respectively, and the average prospect value is denoted as $\bar{V_{A}}$:


(5) $$\begin{array}{l} V_{A1}=\pi \left(b\right)\left(T\left(R_{1}\right)-Z\left(I_{0}+Q\right)\right)+\pi \left(1-b\right)\\ \;\;\;\;\;\;\;\;\;\;\;\;\left(T\left(R_{1}+P\right)-Z\left(I_{0}+hk_{1}\pi \left(q\right)L\right)\right) \end{array}$$

(6) $$\begin{array}{l} V_{A2}=\pi \left(b\right)\left(-Z\left(I_{1}+I_{2}+Q+hk_{2}\pi \left(q\right)L\right)\right)+\\ \;\;\;\;\;\;\;\;\;\;\;\;\;\;\pi \left(1-b\right)\left(-Z\left(I_{1}+I_{2}+h\pi \left(q\right)L\right)\right) \end{array}$$

(7) $$\bar{V_{A}}=aV_{A1}+\left(1-a\right)V_{A2}$$

Similarly, the expected prospect values for the two strategies of the private sector in the supervision of a PPP project are represented $V_{B1}$ and $V_{B2}$, respectively, and the average prospect value is denoted as $\bar{V_{B}}$:


(8) $$\begin{array}{l} V_{B1}=\pi \left(a\right)\left(T\left(R_{2}+Q\right)-Z\left(C_{0}\right)\right)+\\ \;\;\;\;\;\;\;\;\;\;\;\;\;\pi \left(1-a\right)\left(T\left(R_{2}+Q\right)-Z\left(C_{0}+k_{2}\pi \left(q\right)L\right)\right) \end{array}$$

(9) $$\begin{array}{l} V_{B2}=\pi \left(a\right)\left(T\left(R_{3}\right)-Z\left(C_{1}+C_{2}+P+k_{1}\pi \left(q\right)L\right)\right)+\\ \;\;\;\;\;\;\;\;\;\;\;\;\;\;\pi \left(1-a\right)\left(T\left(R_{3}\right)-Z\left(C_{1}+C_{2}+\pi \left(q\right)L\right)\right) \end{array}$$

(10) $$\bar{V_{B}}=bV_{B1}+\left(1-b\right)V_{B2}$$

According to Equation (7) and Equation (10), the replicator dynamics equation of the public sector and the private sector can be described as follows, respectively.


(11) $$\begin{array}{c} F\left(a\right)=\frac{da}{dt}=a\left(V_{A1}-\bar{V_{A}}\right)=a\left(1-a\right)\;\;\left(V_{A1}-V_{A2}\right)\\ =a\left(1-a\right)\left\{\begin{array}{l} \pi \left(b\right)\left[\begin{array}{l} T\left(R_{1}\right)-Z\left(I_{0}+Q\right)\\ +Z\left(I_{1}+I_{2}+Q+hk_{2}\pi \left(q\right)L\right) \end{array}\right]+\\ \pi \left(1-b\right)\left[\begin{array}{l} T\left(R_{1}+P\right)-Z\left(I_{0}+hk_{1}\pi \left(q\right)L\right)\\ +Z\left(I_{1}+I_{2}+h\pi \left(q\right)L\right) \end{array}\right] \end{array}\right\} \end{array}$$

(12) $$\begin{array}{c} W\left(b\right)=\frac{db}{dt}=b\left(V_{B1}-\bar{V_{B}}\right)=b\left(1-b\right)\left(V_{B1}-V_{B2}\right)\\ \;\;\;\;\;\;\;\;\;\;\;\;\;\;=b\left(1-b\right)\left\{\begin{array}{c} \pi \left(a\right)\left[\begin{array}{l} T\left(R_{2}+Q\right)-Z\left(C_{0}\right)-T\left(R_{3}\right)\\ +Z\left(C_{1}+C_{2}+P+k_{1}\pi \left(q\right)L\right) \end{array}\right]+\\ \pi \left(1-a\right)\;\left[\begin{array}{l} T\left(R_{2}+Q\right)-Z\left(C_{0}+k_{2}\pi \left(q\right)L\right)\\ -T\left(R_{3}\right)+Z\left(C_{1}+C_{2}+\pi \left(q\right)L\right) \end{array}\right] \end{array}\right\} \end{array}$$

Let $H=T\left(R_{1}\right)-Z\left(I_{0}+Q\right)+Z\left(I_{1}+I_{2}+Q+hk_{2}\pi \left(q\right)L\right)$ and $U=T\left(R_{1}+P\right)-Z\left(I_{0}+hk_{1}\pi \left(q\right)L\right)+Z\left(I_{1}+I_{2}+h\pi \left(q\right)L\right)$, Equation (11) can be simplified to


(13) F(a)=a(1−a)[π(b)H+π(1−b)U]

where H denotes the difference between the public sector’s payoff of strong supervision and weak supervision when the private sector’s strategy is $B_{1}$. Urepresents the difference between the public sector’s payoff of strong supervision and weak supervision when the private sector’s strategy is $B_{2}$.

Let $E=T\left(R_{2}+Q\right)-Z\left(C_{0}\right)-T\left(R_{3}\right)+Z\left(C_{1}+C_{2}+P+k_{1}\pi \left(q\right)L\right)$ and $G=T\left(R_{2}+Q\right)-Z\left(C_{0}+k_{2}\pi \left(q\right)L\right)-T\left(R_{3}\right)+Z\left(C_{1}+C_{2}+\pi \left(q\right)L\right)$, Equation (12) can be simplified to


(14) W(b)=b(1−b)[π(a)E+π(1−a)G]

where E denotes the difference between the private sector’s payoff under the fulfilling contract strategy and the opportunistic behavior strategy when the public sector’s strategy is $A_{1}$. G represents the difference between the private sector’s payoff under the fulfilling contract strategy and the opportunistic behavior strategy when the public sector’s strategy is $A_{2}$.

### 4.2. Stability analysis of the equilibriums

Let *F*(a) and *F*(p) equal to 0, respectively *W*(b), *F*(q) = (0,0), five equilibrium points of the evolutionary game system: $M_{1}\left(0,0\right),$ $M_{2}\left(0,1\right),$ $M_{3}\left(1,0\right),$ $M_{4}\left(1,1\right),$ and $M_{5}\left({\left(\frac{G}{G-E}\right)}^{\frac{1}{r}},{\left(\frac{U}{U-H}\right)}^{\frac{1}{r}}\right)$ can be obtained. The five equilibrium points are not necessarily the system’s evolutionary stability strategy (ESS), so it is necessary to determine whether they are stable. Friedman (1998) argued that the ESS could be obtained by stability analysis of the system’s Jacobian matrix. According to Equation (13) and Equation (14), the Jacobian matrix can be expressed as follows:


$$\begin{array}{c} J=\left[\begin{array}{cc} \frac{\partial F\left(a\right)}{\partial a} & \frac{\partial F\left(a\right)}{\partial b}\\ \frac{\partial W\left(b\right)}{\partial a} & \frac{\partial W\left(b\right)}{\partial b} \end{array}\right]\\ =\left[\begin{array}{cc} \left(1-2a\right)\left[\pi \left(b\right)H+\pi \left(1-b\right)U\right] & a\left(1-a\right)\left[\frac{d\pi \left(b\right)}{db}H+\frac{d\pi \left(1-b\right)}{db}U\right]\\ b\left(1-b\right)\left[\frac{d\pi \left(a\right)}{da}E+\frac{d\pi \left(1-a\right)}{da}G\right] & \left(1-2b\right)\left[\pi \left(a\right)E+\pi \left(1-a\right)G\right] \end{array}\right] \end{array}$$

(15) $$J=\left[\begin{array}{cc} \frac{\partial F\left(a\right)}{\partial a} & \frac{\partial F\left(a\right)}{\partial b}\\ \frac{\partial W\left(b\right)}{\partial a} & \frac{\partial W\left(b\right)}{\partial b} \end{array}\right]=\left[\begin{array}{cc} F_{11} & F_{12}\\ F_{21} & F_{22} \end{array}\right]$$

where each element in the Jacobian matrix can be expressed as follows:


$$\{\begin{array}{c} F_{11}=\left(1-2a\right)\left[\pi \left(b\right)H+\pi \left(1-b\right)U\right]\\ \;F_{12}=a\left(1-a\right)\left[\frac{d\pi \left(b\right)}{db}H+\frac{d\pi \left(1-b\right)}{db}U\right]\\ F_{21}=b\left(1-b\right)\left[\frac{d\pi \left(a\right)}{da}E+\frac{d\pi \left(1-a\right)}{da}G\right]\\ F_{22}=\left(1-2b\right)\left[\pi \left(a\right)E+\pi \left(1-a\right)G\right] \end{array}$$

The determinant of the Jacobian matrix is as follows:


(16) $$\begin{array}{l} \det\left(J\right)=F_{11}F_{22}-F_{12}F_{21}\\ \begin{array}{l} \;\;\;\;\;\;\;\;\;\;\;\;\;\;\;\;=\left(1-2a\right)\left[\pi \left(b\right)H+\pi \left(1-b\right)U\right]\\ \;\;\;\;\;\;\;\;\;\;\;\;\;\;\;\;\;\;\;\;\left(1-2b\right)\left[\pi \left(a\right)E+\pi \left(1-a\right)G\right] \end{array}\\ \begin{array}{l} \;\;\;\;\;\;\;\;\;\;\;\;\;\;\;\;\;\;\;-a\left(1-a\right)\left[\frac{d\pi \left(b\right)}{db}H+\frac{d\pi \left(1-b\right)}{db}U\right]\\ \;\;\;\;\;\;\;\;\;\;\;\;\;\;\;\;\;\;\;b\left(1-b\right)\left[\frac{d\pi \left(a\right)}{da}E+\frac{d\pi \left(1-a\right)}{da}G\right] \end{array} \end{array}$$

The trace of the Jacobian matrix is expressed as follows:


(17) $$\begin{array}{l} tr\left(J\right)=F_{11}+F_{22}=\left(1-2a\right)\left[\pi \left(b\right)H+\pi \left(1-b\right)U\right]+\\ \;\;\;\;\;\;\;\;\;\;\;\;\;\;\;\;\;\;\left(1-2b\right)\left[\pi \left(a\right)E+\pi \left(1-a\right)G\right] \end{array}$$

When the matrix satisfies det(J)>0, tr(J)<0, the equilibrium point is the ESS (Su, 2020). $M_{1}$, $M_{2}$, $M_{3}$, and $M_{4}$ are the boundary points of the evolutionary game. The area M surrounded by these points denotes the domain of equilibrium solution for the game model between the public sector and the private sector. Accordingly, M={(a,b)|0≤a≤1,0≤b≤1}. Due to G>G−E and U>U−H_,_ the value of $M_{5}$ is greater than 1. $M_{5}$ is not in the area of M, therefore, we only discussed the asymptotic stability of $M_{1}$, $M_{2}$, $M_{3}$, and $M_{4}$. The stability analysis of these equilibrium points is shown in Table 4.

**Table 4:** The stability analysis of the equilibrium point.

Constrain conditions	The equilibrium point	*det* (*J*)	Symbol	*tr* (*J*)	Symbol	Stability
U>0,G>0and H>0,E>0	$M_{1}\left(0,0\right)$	*UG*	+	*U* + *G*	+	Unstable point
	$M_{2}\left(0,1\right)$	*–HG*	–	*H* – *G*	Unsure	Saddle point
	$M_{3}\left(1,0\right)$	*–UE*	–	*E* – *U*	Unsure	Saddle point
	$M_{4}\left(1,1\right)$	*HE*	+	–(*H* + *E*)	–	Stable point
U<0,G<0and H<0,E<0	$M_{1}\left(0,0\right)$	*UG*	+	*U* + *G*	–	Stable point
	$M_{2}\left(0,1\right)$	*–HG*	–	*H* – *G*	Unsure	Saddle point
	$M_{3}\left(1,0\right)$	*–UE*	–	*E* – *U*	Unsure	Saddle point
	$M_{4}\left(1,1\right)$	*HE*	+	–(*H* + *E*)	+	Unstable point
U>0,E<0	$M_{1}\left(0,0\right)$	*UG*	Unsure	*U* + *G*	Unsure	Saddle point
	$M_{2}\left(0,1\right)$	*–HG*	Unsure	*H* – *G*	Unsure	Saddle point
	$M_{3}\left(1,0\right)$	*–UE*	+	*E* – *U*	–	Stable point
	$M_{4}\left(1,1\right)$	*HE*	Unsure	–(*H* + *E*)	Unsure	Saddle point
G>0,H<0	$M_{1}\left(0,0\right)$	*UG*	Unsure	*U* + *G*	Unsure	Saddle point
	$M_{2}\left(0,1\right)$	*–HG*	+	*H* – *G*	–	Stable point
	$M_{3}\left(1,0\right)$	*–UE*	Unsure	*E* – *U*	Unsure	Saddle point
	$M_{4}\left(1,1\right)$	*HE*	Unsure	–(*H* + *E*)	Unsure	Saddle point

It can be seen from Table 4 that the evolutionary game system reaches the ideal equilibrium state at $M_{4}$ when U>0,G>0,H>0, and E>0. It means both the public and private sectors will take active behaviors when the valence perceptions of their active behaviors are more significant than their negative behaviors, and the cost perceptions of their active behaviors are lower than that of their negative behaviors. However, in reality, some factors prevent the system from reaching the optimal state, such as high-cost perception of active behavior by game players, game players’ bounded rationality making their decisions subjective or overconfident, and game players’ different risk attitudes and reference points, et al. (Wu et al., 2020; Zhao et al., 2022).

## 5. Numerical simulation and discussion

We use MATLAB software to simulate the evolution of strategies when different variables change, thereby analyzing the impact of different variables on the strategy of the public and private sectors in the supervision of PPP projects. According to the data of the triple-supply retrofit project in Xinzhuang, Shanghai, and previous studies (Sen and Kitamura, 2004; Van de Kaa, 2010), the initial values of the variables are set as follows: $R_{1}=3$, $I_{0}=3$, $I_{1}=1$, $I_{2}=1$, $R_{2}=3$, $R_{3}=4$, $C_{0}=3$, $C_{1}=1$, $C_{2}=1$, Q=2, P=2, L=10, $k_{1}=0.4$, $k_{2}=0.6$, h=1, q=0.03, $S_{0}=0.9$, $S_{1}=0.9$, λ=2, α=0.88, β=0.88, δ=2, φ=0.98, σ=0.98, r=0.75, a=0.6, and b=0.4. During the simulation process, we change the value of the analyzed parameter with the value of other parameters unchanged. The results of the simulation analysis are shown as follows:

### 5.1. Strategy evolution under different values of $I_{0}$ and $C_{0}$

**Figure 1:**
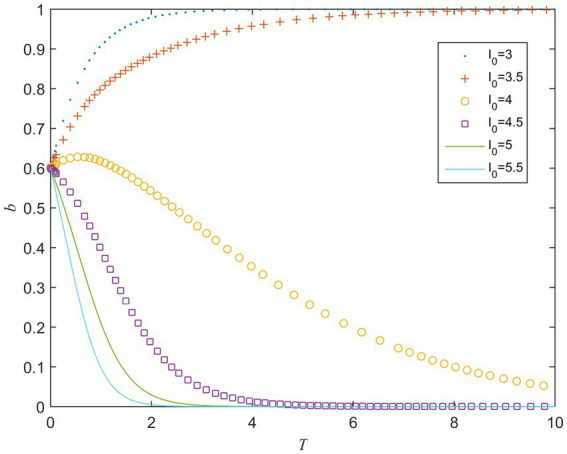
Strategy evolution of the public sector under different values of $I_{0}$.

$I_{0}$ refers to the perceived value of cost for the public sector when its strategy is strong supervision. When the value of $I_{0}$ ranges from 3 to 5.5, the evolution result is shown in Figure 1. It can be seen from Figure 1 that there is a critical value of $I_{0}$ between 3.5 and 4.0 when other parameters remain unchanged. When $I_{0}$ is greater than the critical value, a converges to 0. When it is less than the critical value, a converges to 1. It means that when the public sector adopts a strong supervision strategy, as its perceived value of the cost increases, its strategy gradually evolves to weak supervision, that is, the high supervision cost will hinder the public sector from choosing a strong supervision strategy.

**Figure 2:**
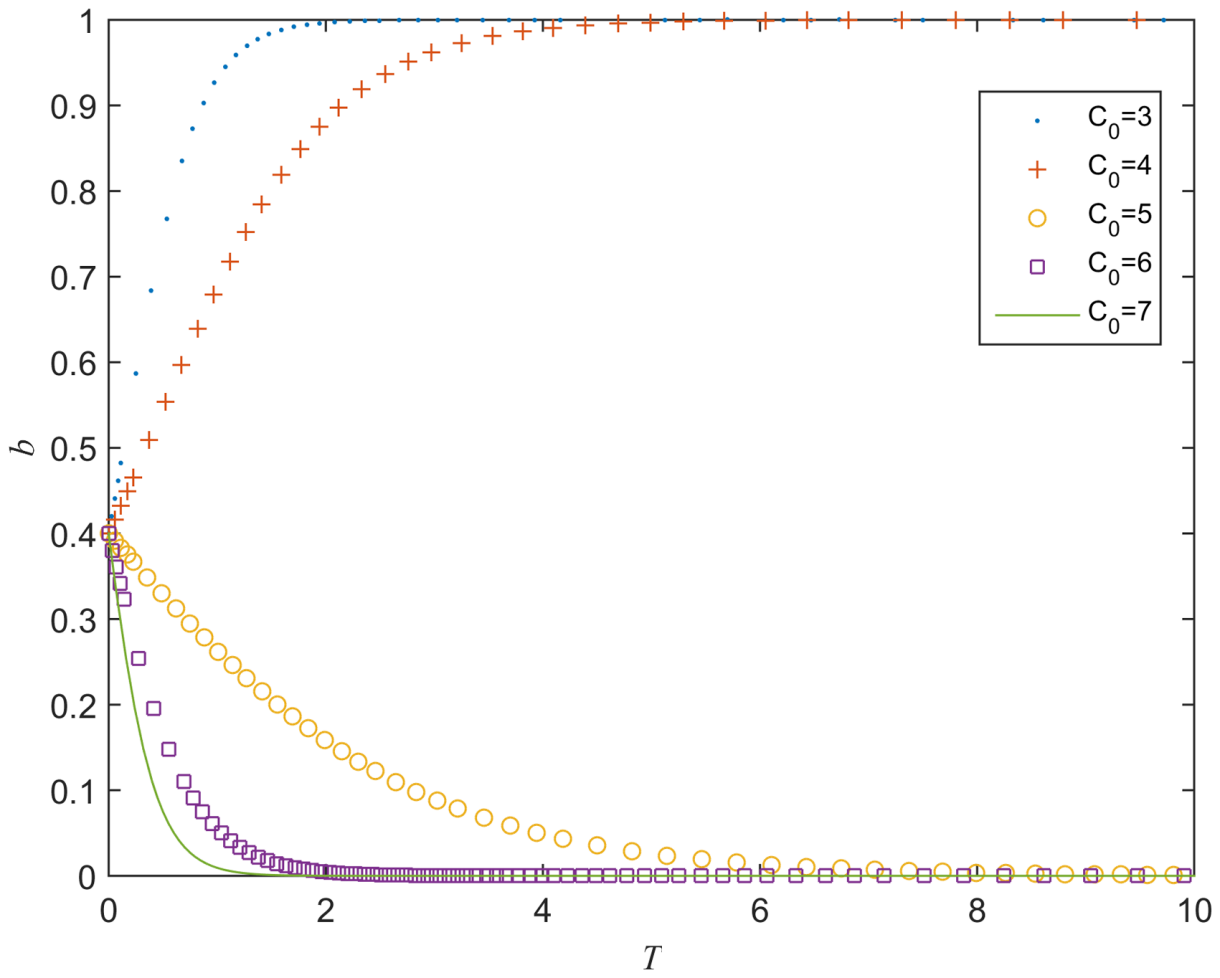
Strategy evolution of the private sector under different values of $C_{0}$.

$C_{0}$ refers to the perceived value of the cost for the private sector when it fulfills contracts. When the value of $C_{0}$ ranges from 3 to 7, the evolution result is shown in Figure 2. It can be seen from Figure 2 that when other parameters remain unchanged, the speed of b converging to 1 becomes slower as the value of $C_{0}$ increases. When $C_{0}$ is greater than a certain critical value, b gradually begins to converge to 0, and the larger $C_{0}$ is, the faster bconverges to 0. It means that when the private sector actively fulfills the contract, as its perceived value of the cost increases, its strategy gradually evolves to opportunistic behavior, that is, the high-cost perception will lead to opportunistic behavior.

The above analysis shows that, whether it is the public sector or the private sector, the increase in perceived value of the cost will hinder their choice of active behavior strategy. Therefore, reducing the players’ cost perception of choosing active behaviors plays a key role in the smooth implementation of PPP projects. This is consistent with the conclusions of Liu et al. (2017).

### 5.2. Strategy evolution under different values of P and Q

**Figure 3:**
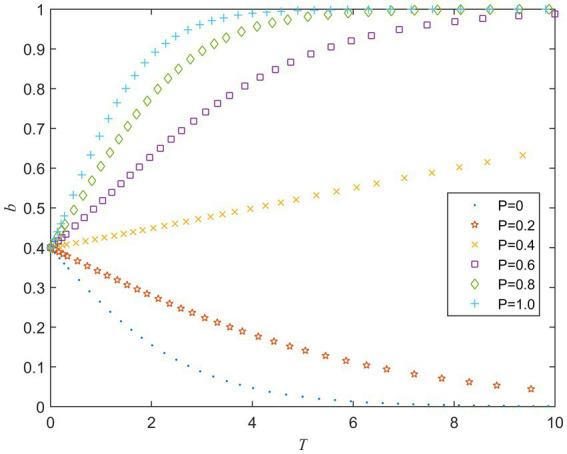
Strategy evolution of the private sector under different values of *P*.

P refers to the penalties for the private sector’s opportunistic behavior. Figure 3 shows the evolution of the private sector’s strategy for different values of P. It can be seen from Figure 3 that when the value of P is less than a certain critical value, b converges to 0, indicating that the incentive effect of weak punishment is not obvious. When P is larger than the certain critical value, b gradually converges to 1. The larger P is, the faster b converges to 1. It means that as the penalties for opportunistic behavior increase, the private sector tends to actively fulfill the contract, that is, increasing the punishment is conducive to motivating the private sector to fulfill the contract actively.

**Figure 4:**
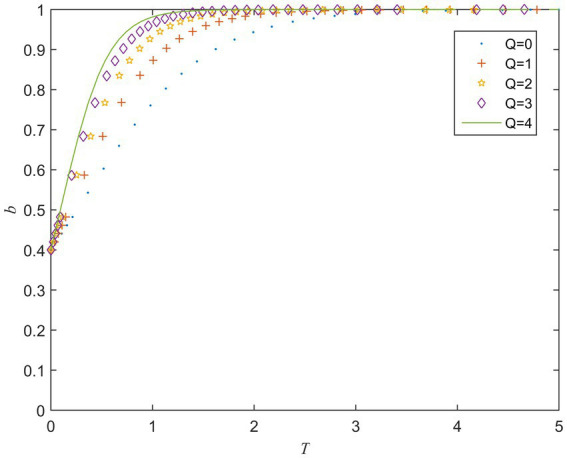
Strategy evolution of the private sector under different values of *Q*.

Q is the reward the private sector receives for actively performing contracts. Figure 4 shows the evolution of the private sector’s strategy for different values of Q. It can be seen from Figure 4 that the larger Q is, the faster b converges to 1. It means that as the incentive for the private sector to actively fulfill the contract increases, the private sector tends to choose an active behavior strategy.

The simulation results show that increasing the rewards for the private sector to choose active behaviors or the punishment for choosing negative behaviors can promote its choice of active strategies. Therefore, setting a reasonable reward and punishment mechanism plays an important role in promoting the private sector to choose an active behavior strategy (Gao and Zhao, 2018). This also was in line with Zhang et al. (2020), who pointed out that by adjusting the coefficient of reward and punishment, the economic benefits of the private sector can be realized, thereby promoting it to fulfill contracts.

### 5.3. Strategy evolution under different values of $S_{0}$and $S_{1}$

**Figure 5:**
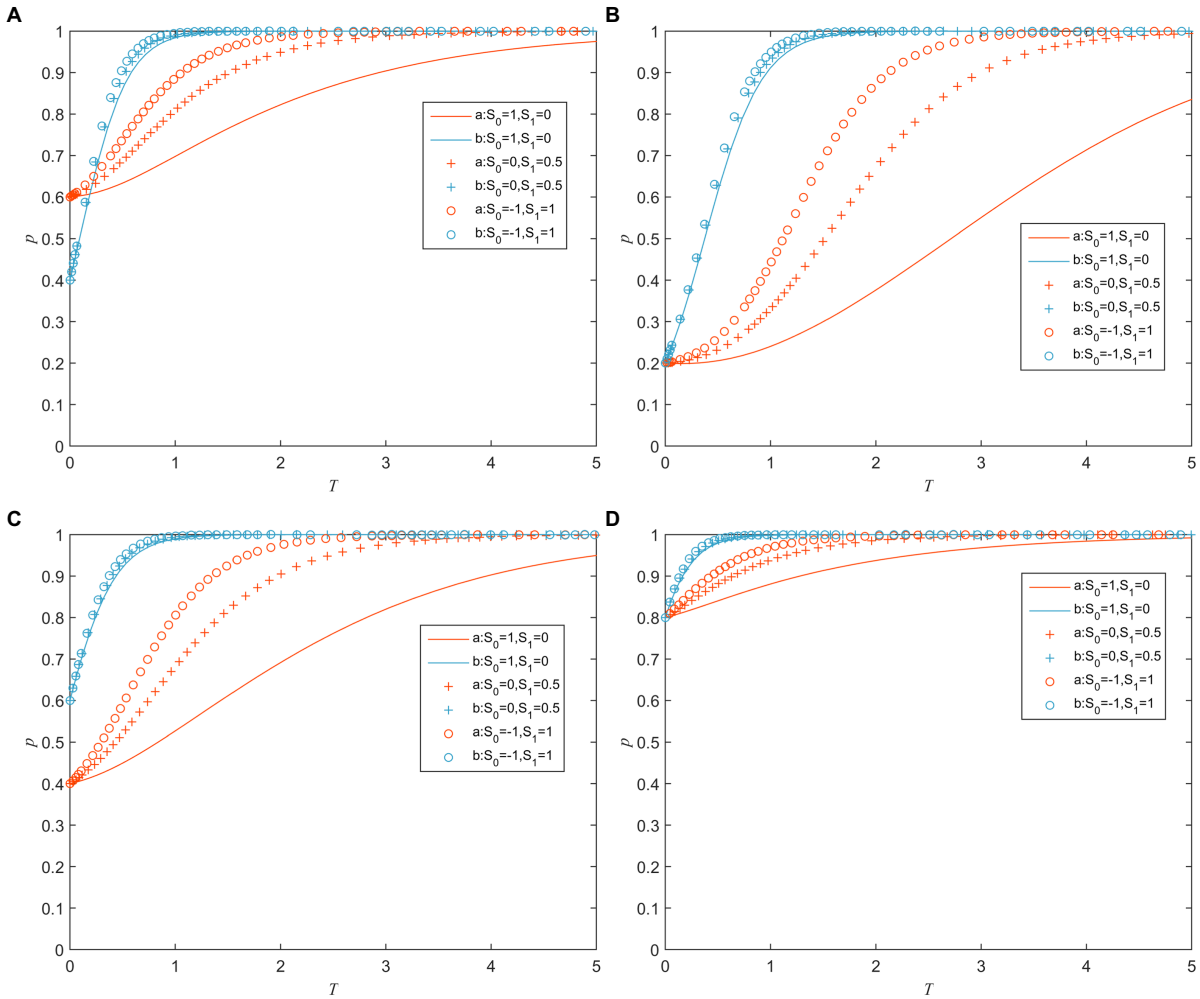
Strategy evolution of game players under different values of $S_{0}$ and $S_{1}$. **(A)** *a* = 0.6 and *b* = 0.4. **(B)** *a* = 0.2 and *b* = 0.2. **(C)** *a* = 0.4 and *b* = 0.6. **(D)** *a* = 0.8 and *b* = 0.8.

$S_{0}$ and $S_{1}$ refer to the reference points in the value function of the valence account and the cost account for the game players’ decision-making behavior, respectively. Figure 5 shows the evolution of the game players’ strategy for different values of $S_{0}$ and $S_{1}$ under different combinations of initial probabilities a and b. As can be seen from Figures 5A–D, although the initial probability values are different, as the value of $S_{0}$ decreases and the value of $S_{1}$ increases, both a and b converge to 1 more quickly. It means that as the values of the cost reference increase and the valence reference decrease, the public sector tends to adopt a strong supervision strategy and the private sector tends to actively fulfill the contract. It is because the increase of the cost reference point will make the player’s perception of loss weaker; the decrease of the valence reference point will make the game player’s perception of the gain stronger. In other words, the players’ perception of valence changes is more significant than cost changes when the values of the cost reference increase and the valence reference decrease.

### 5.4. Strategy evolution under different values of L and h

**Figure 6:**
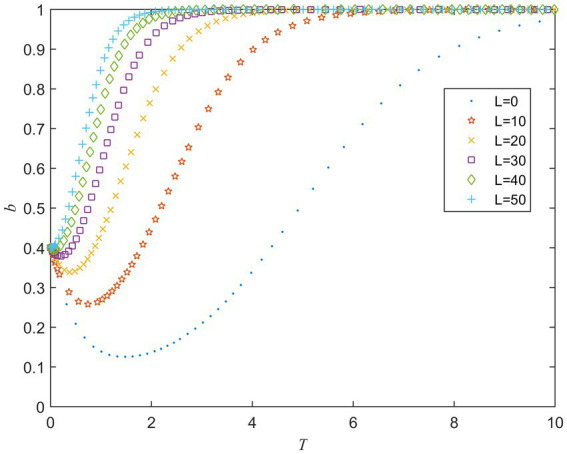
Strategy evolution of the private sector under different values of *L*.

**Figure 7:**
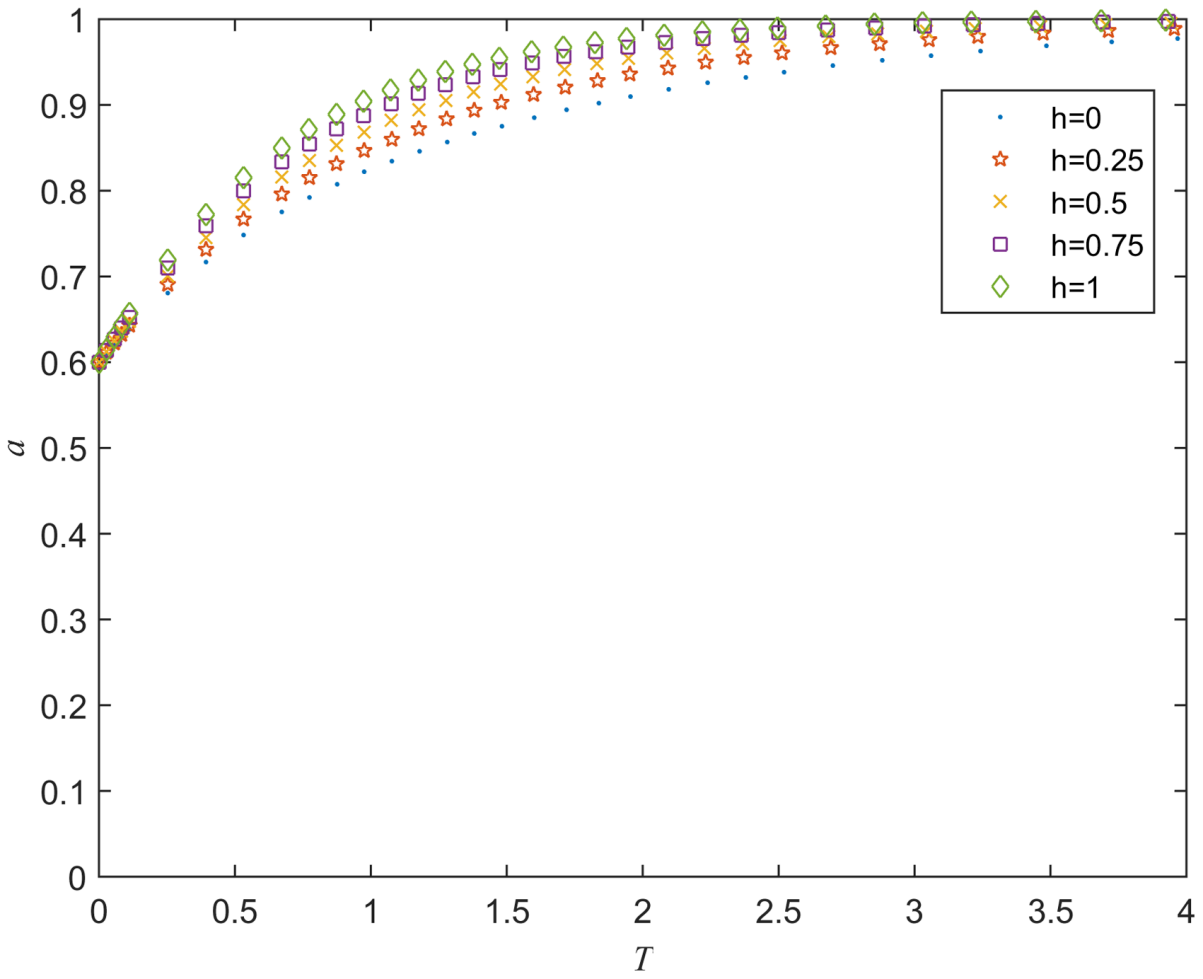
Strategy evolution of the public sector under different values of *h*.

L is the risk loss of the private sector when the strategies of the public and private sector are $\left(A_{2},B_{2}\right)$ and h is the coefficient of joint risk loss. Figure 6 shows the evolution of the private sector’s strategy under different values of L. It can be seen that when the value of L is small, the private sector tends to act opportunistically at first, but eventually tends to fulfill the contract. The private sector is more inclined to take active behavior when the value of L increases. Thus, it can be concluded that a potentially large risk loss will alert the private sector to fulfill contracts actively. Figure 7 shows the evolution of the public sector’s strategy under different values of h. It can be seen from Figure 7 that the public sector is more inclined to strong supervise strategy as the value of h increase. Simulation results suggest that increasing public and private sector’s perceptions of potential risk losses will make them more likely to choose active behavior strategies.

## 6. Conclusion

Considering the public and private sector’s behavioral preferences and value perception in uncertain scenarios, we construct an evolutionary game model for the supervision of PPP projects based on prospect theory, mental accounting theory, and evolutionary game theory. The interactive behavior of the public and private sectors and the equilibrium state of the game system are analyzed. Meanwhile, MATLAB is used for numerical simulation to explore the impact of different variables on their behavioral decisions.

The conclusions of this study are as follows. First, a high-cost perception of supervision may prevent the public sector from adopting strong supervision. A high-cost perception of operations may lead to opportunistic behavior in the private sector. Second, when the penalties for the opportunistic behavior of the private sector are minor, the incentive effect is not apparent. Increasing the penalties for opportunistic behavior of the private sector and the incentives for active behavior will help motivate the private sector to fulfill contracts actively. Third, the increased cost reference points and decreased valence reference points will promote the public and private sectors to take active behavior. Fourth, the public and private sectors are more inclined to take active action when they perceive the potential risk losses to be significant.

Based on the above four conclusions, four corresponding implications and suggestions for the supervision of PPP projects can be put forward as follows.

1. For the public sector, the supervision efficiency should be improved to reduce supervision costs so that the public sector can supervise the private sector powerfully and effectively. Ways to improve supervision efficiency include enhancing the literacy of supervisors, strengthening the supervisory capabilities, using information means, and encouraging different supervision departments to collaborate in the supervision of PPP projects. For the private sector, it can be incentivized to reduce the cost of fulfilling contracts through management and technological innovation.
2. To better motivate the private sector to fulfill contracts actively, the public sector needs to establish a reasonable reward and punishment mechanism, including designing effective reward and punishment schemes, constructing a reasonable payment mechanism, and clarifying the punishment rules for low-quality services.
3. The cost reference points should be increased, and the valence reference points should be decreased to promote active supervision in the public sector and fulfilling contracts in the private sector. In the supervision of PPP projects, the public sector can guide the private sector to reduce the expectation of benefits and actively perform the contract from the perspective of value perception.
4. According to the findings, a high-risk loss perception may motivate the public and private sectors to adopt active behavior. Therefore, the public sector should clearly define the private sector’s liability for contract breach and appropriately increase the penalties, thereby increasing the private sector’s perception of risk losses. In addition, managers can be encouraged to actively supervise by establishing a supervisory responsibility system and clarifying the joint risk responsibilities of the public sector.

The main contributions of this study are as follows. First, we introduce PT-MA into the evolutionary game theory and expand the application of the evolutionary game theory in PPP projects. Compared with previous studies, the combination of prospect theory and mental accounting in this study can provide new ideas for analyzing the behavior of the public and private sectors in the supervision of PPP projects from the perspective of psychological perception. The proposed method enriches the body of knowledge in PPP project supervision. It can also provide a reference for studying supervision issues in other fields. Second, we use prospect theory to reflect the behavioral characteristics of game players when making decisions and classify the value function into a valence account and a cost account according to the mental accounting theory. Accordingly, we construct a payoff matrix based on PT-MA, which can more realistically depict the decision-making behaviors of the public and private sectors in the supervision of PPP projects. Third, we find the influence mechanism of some variables on the decision-making behaviors of the public and private sectors and accordingly put forward some suggestions for effective supervision of PPP projects.

However, the study still has limitations due to specific assumptions. In reality, the decision-makers often face a complex environment in the supervision of PPP projects. Therefore, deepening the model proposed in this study in combination with the actual situation will be further work in the future. In addition, this study only considers the game relationship between the public sector and the private sector, and the public can also be included in the future study.

## Data availability statement

The original contributions presented in the study are included in the article/supplementary material, further inquiries can be directed to the corresponding author.

## Author contributions

XC conceived the initial idea, designed the study, and drafted the original manuscript. MC designed the study, revised the manuscript, and provided the funding support. All authors listed have made a substantial, direct, and intellectual contribution to the work and approved it for publication.

## Conflict of interest

The authors declare that the research was conducted in the absence of any commercial or financial relationships that could be construed as a potential conflict of interest.

## Publisher’s note

All claims expressed in this article are solely those of the authors and do not necessarily represent those of their affiliated organizations, or those of the publisher, the editors and the reviewers. Any product that may be evaluated in this article, or claim that may be made by its manufacturer, is not guaranteed or endorsed by the publisher.
